# An Initial Validation of Community-Based Air-Conduction Audiometry in Adults With Simulated Hearing Impairment Using a New Web App, DigiBel: Validation Study

**DOI:** 10.2196/51770

**Published:** 2024-01-25

**Authors:** Anna Sienko, Arun James Thirunavukarasu, Tanya Kuzmich, Louise Allen

**Affiliations:** 1 School of Clinical Medicine University of Cambridge Cambridge United Kingdom; 2 Oxford University Clinical Academic Graduate School University of Oxford Oxford United Kingdom; 3 Department of Ophthalmology Cambridge University Hospitals NHS Foundation Trust Cambridge United Kingdom; 4 Department of Paediatrics University of Cambridge Cambridge United Kingdom

**Keywords:** audiology, audiometry, hearing test, eHealth, mobile application, automated audiometry, hearing loss, hearing impairment, web-app, web-apps, web-application, digital health, hearing, adult, adults, mobile health, mhealth, community-based, home-based, assistive technology, screening, usability, ears, ear

## Abstract

**Background:**

Approximately 80% of primary school children in the United States and Europe experience glue ear, which may impair hearing at a critical time for speech acquisition and social development. A web-based app, DigiBel, has been developed primarily to identify individuals with conductive hearing impairment who may benefit from the temporary use of bone-conduction assistive technology in the community.

**Objective:**

This preliminary study aims to determine the screening accuracy and usability of DigiBel self-assessed air-conduction (AC) pure tone audiometry in adult volunteers with simulated hearing impairment prior to formal clinical validation.

**Methods:**

Healthy adults, each with 1 ear plugged, underwent automated AC pure tone audiometry (reference test) and DigiBel audiometry in quiet community settings. Threshold measurements were compared across 6 tone frequencies and DigiBel test-retest reliability was calculated. The accuracy of DigiBel for detecting more than 20 dB of hearing impairment was assessed. A total of 30 adults (30 unplugged ears and 30 plugged ears) completed both audiometry tests.

**Results:**

DigiBel had 100% sensitivity (95% CI 87.23-100) and 72.73% (95% CI 54.48-86.70) specificity in detecting hearing impairment. Threshold mean bias was insignificant except at 4000 and 8000 Hz where a small but significant overestimation of threshold measurement was identified. All 24 participants completing feedback rated the DigiBel test as good or excellent and 21 (88%) participants agreed or strongly agreed that they would be able to do the test at home without help.

**Conclusions:**

This study supports the potential use of DigiBel as a screening tool for hearing impairment. The findings will be used to improve the software further prior to undertaking a formal clinical trial of AC and bone-conduction audiometry in individuals with suspected conductive hearing impairment.

## Introduction

Over 5% of the world’s population, approximately 430 million people worldwide, have disabling hearing loss; 34 million of these are children [1]. The main causes of hearing loss in adulthood are age-related hearing loss, noise-related hearing loss, and hearing loss due to chronic otitis media. In children, the most common cause of hearing impairment is otitis media with effusion, also known as “glue ear”. Approximately 80% of primary school children in the United States and Europe experience glue ear [2]. In contrast to most forms of adult hearing loss, hearing impairment in children with glue ear fluctuates. Serial testing is often required to detect and manage the condition to mitigate its adverse impact on social development. Children in low- and middle-income countries, families experiencing socioeconomic deprivation, and disadvantaged populations are disproportionally affected by conductive hearing loss caused by glue ear and its complications, or from damage to the eardrum as seen in chronic tympanic perforations or chronic serous otitis media [3,4]. Since delayed recognition and management of childhood hearing impairment have long-term consequences for socialization and educational attainment, screening audiometry is recommended in primary school [5]. However, population screening programs are hindered by cost, standardization, requirement for staff training, false-positive referrals, and poor data capture [6,7]. Even where available, school screening may miss children with fluctuating hearing loss due to glue ears. Additionally, several year groups have missed screening during the COVID pandemic [8]. These children may face months of impaired hearing before diagnosis and management due to backlogs in audiometry and specialist services.

Hearing thresholds are assessed using pure tone audiometry (PTA) with air-conduction (AC) headphones and bone-conduction (BC) transducers. This usually requires specialist equipment and trained clinicians. Automated audiometry and, more recently, validated self-testing hearing software apps, may improve the accessibility of screening and threshold audiometry testing, particularly in rural areas. The DigiBel web app is a recently developed Class 1 CE marked medical device that enables self-testing of AC and BC hearing levels. Like some other audiometry apps, it is suitable for community use in adults and children without clinical support. DigiBel has the novel facility to undertake BC audiometry with the same transducer used in a BC hearing assistance kit (BC headphones with Bluetooth-connected microphone, Raspberry Pi, Cambridge, United Kingdom). This could identify children who may benefit from this assistive technology while waiting for diagnosis, spontaneous resolution, or definitive management of their glue ear [9].

The purpose of this study of DigiBel audiometry is to determine the app’s sensitivity and specificity for detecting simulated conductive hearing impairment of more than 20 dB and to identify software modifications required prior to formal trials in a clinical population. Given the long protocol of testing or retesting and the requirement for usability feedback to inform improvement in the app design, this preliminary study involved healthy adult volunteers rather than children.

## Methods

### Overview

Healthy adult volunteers from the community without a previous history of hearing impairment were invited to participate in this comparative study of automated PTA and DigiBel audiometry. After receiving an explanation of the study, participants provided verbal consent to proceed to audiometry testing. Each participant was assigned a unique study identification number; no personal identifiable information was recorded.

Testing was undertaken in community settings such as participants’ homes and classrooms by nonaudiologist technicians. Prior to testing, each volunteer was instructed to place a foam earplug firmly into their left auditory canal and requested not to adjust it until testing was complete. This simulated a conductive hearing loss in the plugged ear, making each ear an independent entity for the purpose of statistical analysis and providing a range of hearing levels.

The reference automated AC PTA and the index DigiBel audiometry test were undertaken sequentially in random order. DigiBel audiometry for 4 frequencies was repeated immediately after the initial test, to assess within-session test-retest (TRT) reliability. After completing both audiometry tests, each volunteer was asked to complete a feedback questionnaire covering test preference and usability (Multimedia Appendix 1).

### Automated Pure Tone Audiometry—The Gold-Standard Reference Test

Automated PTA was undertaken using an Oscilla (Oscilla A/S Aarhus Denmark) USB300 audiometer with TDH-39 headphones. The modified Hughson-Westlake algorithm was used for determining the reference AC audiometric threshold [10]. After an initial explanation by the technician and a conditioning test at 1000 Hz, thresholds were recorded at 2000, 4000, 8000, 1000, 500, and 250 Hz in accordance with British Society of Audiology recommendations [11]. The hearing threshold criterion for each frequency was determined as the lowest intensity at which participants accurately signaled 2 confirmations out of 3 presentations. The number of false positive responses was manually recorded.

### The DigiBel Index Test

DigiBel has been laboratory and biologically calibrated (Institute of Sound and Vibration Research, University of Southampton, United Kingdom; and Chears-audiology, Royston, United Kingdom) to run specifically on any model iPad tablet (Apple) with Sennheiser HD 400S AC headphones (Wedemark, Germany).

A pretest embedded video provides instructions for use and a checklist ensures that the iPad volume is on maximum and the headphones are fitted correctly. Noise sampling, using the inbuilt Sennheiser headphone microphone, ensures that the ambient noise level is less than 40 dB prior to testing. Testing can be paused and restarted at any stage if the ambient noise level changes unexpectedly or interruptions occur. Configurable settings include a choice of warble or pure tone, test frequencies, ambient noise setting, and child or adult version of the test.

**Figure 1:**
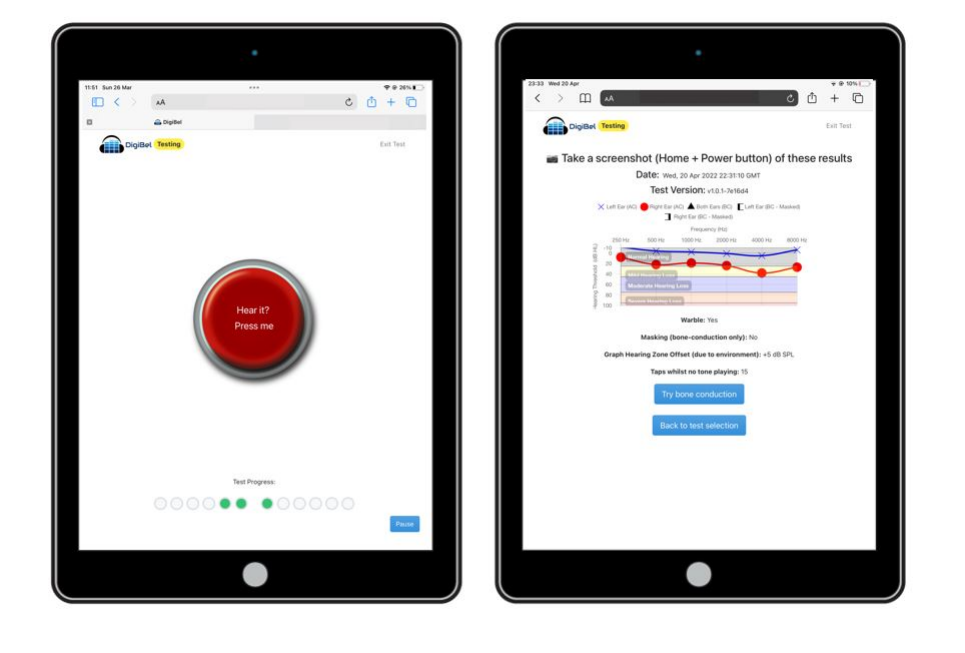
(A) DigiBel test interface and (B) DigiBel audiometry graph.

The user taps a central animated button on the iPad display when they hear the tone (Figure 1A). A conditioning step requires the user to accurately tap the button on hearing a random onset suprathreshold tone at 1000 Hz before testing can begin. During testing, the onset of the tone is randomized from 0 to 3 seconds after the appearance of the response button to avoid a predictable response pattern. The tone stops in response to the tap and a psychophysical staircase algorithm starting from 60 dB (10 dB down, 5 dB up) is followed in a 2 down, 1 up rule, requiring 5 reversals dependent on the user’s input. The final threshold is calculated as the mean of the final 3 reversal thresholds. Once completed, a standard audiometry graph and the number of false positive responses are displayed (Figure 1B). The user can choose to repeat testing or undertake BC testing to determine the functional effect on hearing levels.

For this study, DigiBel AC PTA was undertaken in the sequence 2000, 4000, 8000, 1000, 500, and 250 Hz and retested in the sequence 2000, 4000, 8000, and 500 Hz using the adult test version.

### Statistics

Data were analyzed in R (version 4.1.2; R Foundation for Statistical Computing) [12,13]. Accuracy and TRT reliability were assessed through Bland-Altman analysis, a statistical approach enabling analysis of the agreement between 2 measurement methods by assessing their mean differences (mean bias) and upper and lower limits of agreement (SD 1.96). Qualitative appraisal of mixed effects model 2-way intraclass correlation coefficients (ICCs) between the threshold measurements from each device was based on conventional standards with moderate, good, and excellent agreement indicated by an ICC of ≥0.50, ≥0.75, and ≥0.90, respectively [14]. The correlation between calculated bias and mean threshold values was analyzed using Pearson correlation coefficients (PCCs). The percentages of DigiBel threshold measurements lying within 10 dB of both the reference test and the repeated DigiBel test were calculated [15,16]. Statistical significance was calculated for the mean bias where confidence intervals did not cross zero, and for ICC and PCCs where *P*<.05. To assess diagnostic efficacy, sensitivity and specificity for detection of hearing thresholds above 20 Hz were calculated, using automated PTA as the reference. The Student *t* test was used to compare the number of false positive responses for each test. Throughout, magnitudes were reported as the mean (SD) unless otherwise stated.

### Ethical Considerations

The study adhered to the tenets of the Declaration of Helsinki and the protocol was approved by the Quality and Safety Committee of Cambridge University Hospitals National Health Service Foundation Trust as part of a service improvement project. The Quality and Safety Committee at Cambridge University Hospitals considered that formal ethics committee approval was not required for this no-risk service improvement study in a healthy, adult population and opined that verbal consent was sufficient in these circumstances to prevent the collection of patient identifiable information (approval for Service Evaluation Project – PRN9288; dated April 11, 2023). No other demographic information other than age was recorded to maintain anonymity for this service improvement study. No compensation was offered for participating in the study.

## Results

### Participant Characteristics

**Figure 2:**
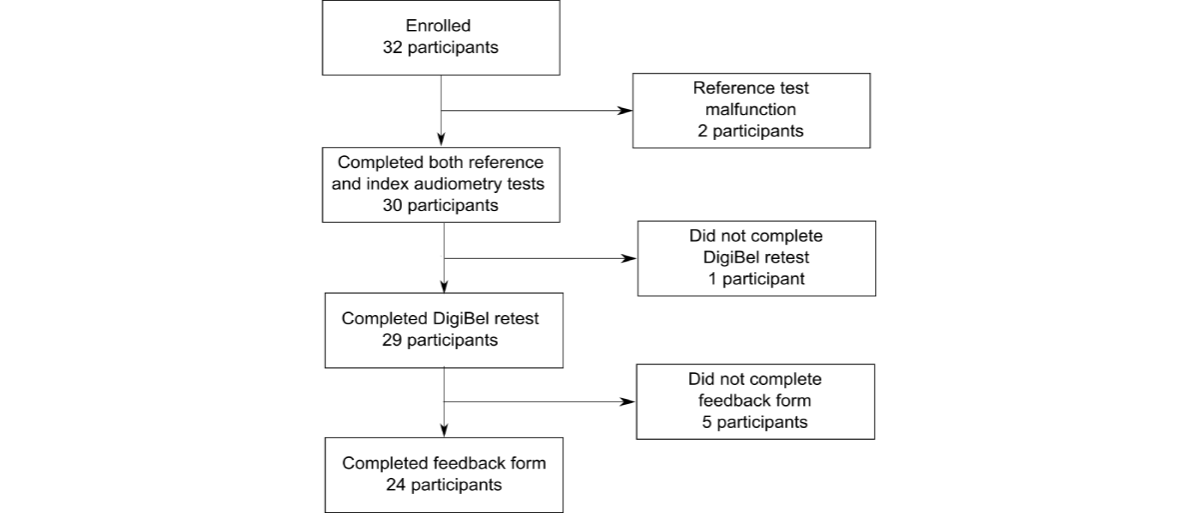
Participant flow diagram.

A total of 32 healthy participants agreed to take part, but 2 participants were excluded due to malfunction of the reference automated PTA test. The 30 participants who completed both the reference and index tests were 21-66 (mean 27.9, SD 10.3) years. TRT data were collected from 29 participants (one volunteer left early due to time constraints). Feedback forms were completed by 24 participants (Figure 2).

### Accuracy and Reliability of DigiBel

**Figure 3:**
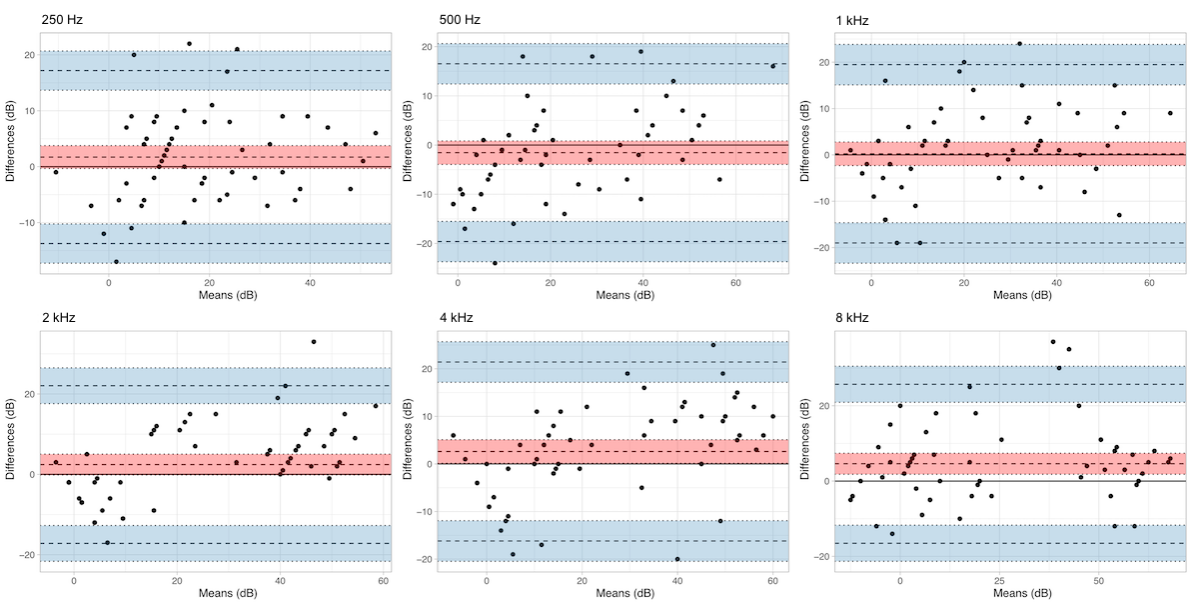
Comparison of DigiBel and automated pure tone audiometry using Bland-Altman plots at 6 frequencies. Bland-Altman plots comparing mean and difference in threshold measurements (in dB) for DigiBel and standard automated pure tone audiometry at 6 frequencies (in Hz). The 95% CIs are shaded for the bias (red) and 95% limits of agreement (blue).

Across the 6 tested frequencies, threshold hearing levels of DigiBel compared with automated PTA gave an ICC of above 0.75 (good or excellent agreement) and *P*<.001 in every trial (Table 1; Multimedia Appendices 2 and 3). The mean lower limit of agreement (LOA) was –17.04 dB (SD 2.12 dB) and the mean upper LOA was 20.39 dB (SD 3.41 dB). No significant bias was apparent except at 4000 and 8000 Hz where a small but statistically significant bias was apparent (2.62 and 4.60 dB, respectively), with DigiBel providing systematically higher threshold results at those frequencies (Figure 3). An agreeable level of threshold difference in audiometry assessments has previously been defined as 10 dB; 263 out of 360 (73%) of DigiBel threshold measurements were within this standard [16]. There was a significant positive PCC between the measurement bias and mean at 500 (*P*<.001), 1000 (*P*=.005), 2000 (*P*<.001), and 4000 Hz (*P*<.001) test frequencies.

**Table 1:** Comparison of threshold values (in dB) using Bland-Altman statistics and intraclass correlation coefficients.

Comparison and frequency (Hz)		Number of ears tested	Bias (dB), 95% CI	LLoA^a^ (dB), 95% CI	ULoA^b^ (dB), 95% CI	ICC^c^, 95% CI	PCC^d^ (*r*)
**DigiBel compared with standard automated audiometry^e^**							
	250	60	1.72 (–0.32 to 3.76)	–13.75 (–17.25 to –10.25)	17.18 (13.68 to 20.69)	0.85 (0.77 to 0.91)	0.16
	500	60	–1.55 (–3.93 to 0.83)	–19.62 (–23.71 to –15.52)	16.52 (12.42 to 20.61)	0.87 (0.80 to 0.92)	0.48
	1000	60	0.23 (–2.30 to 2.77)	–19.01 (–23.36 to –14.65)	19.47 (15.11 to 23.83)	0.88 (0.80 to 0.92)	0.36
	2000	60	2.43 (–0.15 to 5.02)	–17.18 (–21.62 to –12.74)	22.05 (17.61 to 26.49)	0.88 (0.81 to 0.93)	0.64
	4000	60	2.62 (0.14 to 5.10)	–16.19 (–20.45 to –11.93)	21.42 (17.16 to 25.68)	0.89 (0.83 to 0.94)	0.48
	8000	60	4.60 (1.82 to 7.38)	–16.51 (–21.29 to –11.73)	25.71 (20.93 to 30.49)	0.91 (0.82 to 0.95)	0.13
**DigiBel test-retest comparison^e^**							
	500	58	0.26 (–1.91 to 2.43)	–15.90 (–19.62 to –12.17)	16.42 (12.69 to 20.14)	0.92 (0.87 to 0.95)	N/A^f^
	2000	58	1.40 (–0.65 to 3.44)	–13.84 (–17.36 to –10.33)	16.64 (13.12 to 20.15)	0.95 (0.91 to 0.97)	N/A
	4000	58	0.90 (–0.66 to 2.46)	–10.74 (–13.42 to –8.06)	12.53 (9.85 to 15.22)	0.97 (0.94 to 0.98)	N/A
	8000	58	0.98 (–1.23 to 3.19)	–15.48 (–19.28 to –11.68)	17.44 (13.65 to 21.24)	0.95 (0.92 to 0.97)	N/A

^a^LLoA: lower limit of agreement.

^b^ULoA: upper limit of agreement.

^c^ICC: intraclass correlation coefficient.

^d^PCC: Pearson correlation coefficient (calculated for the relationship between measurement bias and mean for each frequency).

^e^Comparison of threshold values (in dB) using Bland-Altman statistics and ICCs between DigiBel and standard automated PTA and DigiBel test and retest.

^f^N/A: not applicable.

In TRT comparisons, ICC was above 0.90 (excellent) at all tested frequencies: 500, 2000, 4000, and 8000 Hz (Table 1). No statistically significant mean bias was exhibited at any frequency. The mean lower LOA was –13.99 dB (SD 2.34 dB); the mean upper LOA was 15.76 dB (SD 2.20 dB). Overall, 85% of TRT thresholds were within 10 dB of each other.

A mean of 3.57 (SD 4.68) false positive responses were recorded during reference testing and 11.43 (SD 7.12) during the first DigiBel test (*P*<.001).

### Sensitivity and Specificity

The sensitivity and specificity of DigiBel for detecting 20 dB hearing loss at each frequency are shown in Table 2. When applied to the 4 frequencies used for screening (250, 1000, 2000, and 4000 Hz), DigiBel had 100% sensitivity (95% CI 87.23-100) and 72.73% (95% CI 54.48-86.70) specificity for detecting 20 dB hearing loss in adults in a quiet setting.

**Table 2:** Screening accuracy of DigiBel for hearing threshold >20 dB identified by automated pure tone audiometry.

Frequency (Hz)	Sensitivity (%), 95% CI	Specificity (%), 95% CI
250	95.00 (75.13 to 99.87)	85.00 (70.16 to 94.29)
500	91.30 (71.96 to 98.93)	89.19 (74.58 to 96.97)
1000	100.00 (86.28 to 100.00)	85.71 (69.74 to 95.19)
2000	100.00 (86.77 to 100.00)	79.41 (62.10 to 91.30)
4000	100.00 (86.28 to 100.00)	88.57 (73.26 to 96.80)
8000	100.00 (86.28 to 100.00)	88.57 (73.26 to 96.80)
Screening frequencies (250, 1000, 2000, and 4000)	100.00 (87.23 to 100.00)	72.73 (54.48 to 86.70)

### Usability

Out of the 24 participants, 21 (88%) participants, while completing the questionnaire, did not regularly use digital health apps. All 24 participants rated DigiBel either good (15/24, 63% of participants) or excellent (9/24, 38% of participants); (7/24, 29% participants preferred the DigiBel test; 6/24, 25% participants preferred the standard test; and 11/24, 46% participants gave no test preference). A total of 21 (88%) participants agreed or strongly agreed that they would be confident to use DigiBel at home without help. The most common qualitative feedback given to the question “what is the best thing about the app?” was that it was easy or intuitive to use (17/24, 71% of participants). Answering “what is the worst thing about the app?” the commonest complaint was that the test was too long or boring (10/24, 42% of participants). One participant commented on environmental noise leaks through the headphones (Multimedia Appendix 1).

## Discussion

### Principal Findings

In 30 healthy adults with simulated unilateral conductive hearing impairment, DigiBel’s screening sensitivity and specificity were 100% (95% CI 87.2-100) and 72.73% (95% CI 54.45-86.7), respectively. The hearing threshold measurement mean bias between DigiBel and automated AC PTA was not significant except at 4000 and 8000 Hz, where it reached statistical significance (2.62 and 4.60 dB higher than the reference, respectively).

At least 5 validated downloadable apps enable automated AC audiometry, several of which support self-testing without clinician involvement [17]. Two apps, uHear (Unitron Ltd) and ShoeBOX (SHOEBOX Ltd) for iOS, include a BC audiometry facility [18,19]. uHear has been calibrated for use with commercial in-ear Apple headphones and ShoeBOX uses purpose-built audiometry headphones. DigiBel is not yet commercialized but its potential advantage is its calibration to use affordable (retail price US $41, equivalent to €39), lightweight, and wipe-clean Sennheiser HD 400S AC headphones. DigiBel supports the use of Raspberry Pi BC headphones (retail price US $28, equivalent to €26) for BC audiometry which may quantify the potential benefit from their use (with a paired microphone) as an assistive technology.

DigiBel’s screening sensitivity and specificity for hearing impairment (more than 20 dB) is comparable to previous studies of both uHear (98.2%-100% sensitivity and 60.0-82.1 specificity) and ShoeBOX (91.2%-93.3% and specificity of 57.8%-94.5%) [18-22]. In this study comparing the threshold measurements of DigiBel to automated AC PTA in 30 individuals, there was a significant positive correlation between the mean bias and the mean threshold measurement at 500, 1000, 2000, and 4000 Hz frequencies, resulting in an overestimation of hearing ability at normal hearing levels and an underestimation of hearing ability in ears with subnormal hearing. This was particularly evident at 2000 Hz testing and may have resulted in the lower specificity demonstrated at this frequency. This is likely to reflect the sound output characteristic of the AC headphones and will require software corrections prior to future clinical studies. Overall, 73% of threshold measurements with DigiBel were within 10 dB of standard PTA; previous studies of ShoeBOX have found over 90% of measurements were within this range [23]. The comparatively poor performance of DigiBel for this metric may be due to environmental noise leakage through the Sennheiser headphones, a disadvantage of their comfort.

Masking of the unplugged ear was not used for either automated PTA or DigiBel because this facility is unlikely to be used by nontrained observers in community settings. A minority of participants in this study had a simulated intra-aural threshold difference exceeding 40 dB. It is possible that intra-aural transmission may have affected threshold values in these individuals, but this would be expected to affect both tests similarly.

The Hughson-Westlake algorithm and other adaptive methods are widely used to assess audiometric threshold, more recently, machine learning techniques have been developed [24]. To our knowledge, DigiBel is the only app to apply a staircase-reversal technique to audiometry, although it is commonly used for visual threshold testing [25]. In this study, the number of false positive participant responses was substantially higher with DigiBel than standard testing. This may be due to the higher number of stimulus presentations compared with the ascending method used in standard automated PTA. The sensitivity of the iPad screen to a tap compared with the standard audiometer’s hand-held responder may be an additional factor. There was no evidence in this study that the higher false positive rate translated into a systematic overestimation of hearing ability.

All participants rated DigiBel as good or excellent, but 42% (n=10) of participants complained that the test took too long or was boring. Test duration was not measured during this study, but threshold testing for 6 frequencies is expected to take approximately 13 minutes with DigiBel, several minutes longer than standard automated PTA, primarily due to its staircase-reversal algorithm. Study participants had performed retesting which may have contributed to the perceived length of testing. The children’s version of the app has cartoons designed to increase interest but, even so, the test duration of threshold audiometry may limit its usability in young children. The DigiBel screening test of 4 frequencies takes approximately 3 minutes and may prove more feasible in this, its target population.

To simulate a range of hearing thresholds in the participant cohort, an earplug was used. This is a major limitation of the study because the effectiveness of the earplug may have altered during testing and earplugs may not accurately mimic genuine hearing impairment. Although this study indicates that DigiBel has acceptable accuracy for detecting simulated hearing impairment in healthy adult volunteers, these results are not generalizable and software corrections are required prior to clinical use.

This preliminary study confirms that the DigiBel app is an acceptable and easy-to-use self-testing web-based tool that accurately detects more than 20 dB of simulated hearing impairment in adults. Minor modifications to the 1000, 2000, and 4000 Hz frequency-specific normalization factors used in the software algorithm which converts sound pressure levels to hearing level are required to ensure uniformity of sound output and accuracy across the range of hearing abilities. A study, conducted in primary school children attending a hospital audiology clinic, is underway to assess the accuracy of DigiBel in identifying conductive hearing impairment. Additionally, the innovative concept behind DigiBel will be tested: its ability to identify those children who could benefit from the temporary use of a BC hearing assistance kit for use at school and home, and the impact this has on quality of life (using a parent- or patient-reported outcome measure questionnaire) while waiting for specialist care.

### Conclusions

The World Health Organization identifies hearing loss as a major global health issue, with two-thirds of people with severe hearing loss living in low and middle countries with poor access both to hearing testing (audiometry) or conventional hearing aids. It can affect many aspects of life such as education, employment, and communication, and result in social isolation.

Several software apps like DigiBel, studied here, have been developed to enable individuals to test their own hearing in the community. Uniquely, DigiBel has the additional potential to identify individuals with hearing loss who could derive immediate hearing support from an affordable and rechargeable bone-conduction hearing assistance kit while waiting for specialist care.

This initial study of DigiBel provides confirmation that the app is easy to use and accurate at detecting simulated hearing impairment. It identifies some software corrections that may improve its accuracy and lays the groundwork for future clinical studies to assess DigiBel’s performance in children and adults with hearing impairment.

## Appendix

Multimedia Appendix 1 Feedback questionnaire.

Multimedia Appendix 2 Feedback data.

Multimedia Appendix 3 Audiometry dataset.

## Abbreviations

AC: air-conduction
BC: bone-conduction
ICC: Intraclass correlation coefficient
LOA: limit of agreement
PCC: Pearson correlation coefficient
PTA: pure tone audiometry
TRT: test-retest
