# Supplementary material for: An Initial Validation of Community-Based Air-Conduction Audiometry in Adults With Simulated Hearing Impairment Using a New Web App, DigiBel: Validation Study
Source: JMIR Form Res. 2024 Jan 25;8:e51770. doi: 10.2196/51770 (PMC10853851; doi:10.2196/51770)
Supplement: Multimedia Appendix 1 [file formative_v8i1e51770_app1.docx]

Study number:

### DIGIBEL FEEDBACK FORM

Please help us to evaluate and improve DigiBel by answering the following questions:

**Your age:**

**Do you regularly use any healthcare (not fitness) apps?**

Yes  No

If yes, which ones have you used? …………………………………

| **Please rate the following:** | Strongly disagree | Disagree | Neutral | Agree | Strongly agree |
| --- | --- | --- | --- | --- | --- |
| The instruction video was easy to follow | 1 | 2 | 3 | 4 | 5 |
| You were able to use the app without help | 1 | 2 | 3 | 4 | 5 |
| You would feel confident using the app at home | 1 | 2 | 3 | 4 | 5 |
| You would find it useful to use the app at home | 1 | 2 | 3 | 4 | 5 |

(if you had a hearing problem)

**Overall, how do you rate the DigiBel hearing test?**

| Poor 🞎 | Fair 🞎 | Good 🞎 | Excellent 🞎 |
| --- | --- | --- | --- |

**Which test did you prefer?** DigiBel 🞎 Standard test 🞎 No preference 🞎

**What was the best thing about the app?**

**What was the worse things about the app?**

**How could we improve the app?**

**Any other comments?**
